# The Use of Virtual Reality in Psychology: A Case Study in Visual Perception

**DOI:** 10.1155/2015/151702

**Published:** 2015-08-03

**Authors:** Christopher J. Wilson, Alessandro Soranzo

**Affiliations:** ^1^Teesside University, Middlesbrough TS1 3BA, UK; ^2^Sheffield Hallam University, Sheffield S10 2BP, UK

## Abstract

Recent proliferation of available virtual reality (VR) tools has seen increased use in psychological research. This is due to a number of advantages afforded over traditional experimental apparatus such as tighter control of the environment and the possibility of creating more ecologically valid stimulus presentation and response protocols. At the same time, higher levels of immersion and visual fidelity afforded by VR do not necessarily evoke presence or elicit a “realistic” psychological response. The current paper reviews some current uses for VR environments in psychological research and discusses some ongoing questions for researchers. Finally, we focus on the area of visual perception, where both the advantages and challenges of VR are particularly salient.

## 1. Introduction

The proliferation of available virtual reality (VR) tools has seen increased use in experimental psychology settings over the last twenty years [1–4]. For the researcher, VR is compelling due to the almost limitless possibilities for the creation of stimuli and this has led to spread of VR into domains such as clinical and developmental psychology, which one might not have initially anticipated [5–7]. Once considered to be an “answer without a question,” VR is now firmly established as an experimental tool [8]. However, in addition to the many advantages associated with the use of VR, there remain some drawbacks and ongoing questions. Of course, the relative importance of these issues is dependent entirely on the use case; while presence may be important in a clinical setting, for example, issues with space perception may limit the accuracy of a physical reach task. Similarly, in the experimental examination of visual perception, potential differences between actual and virtual reality can either be advantageous or detrimental. In this paper we provide a brief overview of the benefits and challenges associated with VR in psychology research and discuss its utility in relation to the examination of visual perception.

The term VR is often used interchangeably to refer to one of three types of system: a virtual environment presented on a flat screen, a room-based system such as a CAVE, or a head-mounted display (HMD: [9, 10]). Though all three systems are quite different, a common feature of all three is the introduction of stereoscopic depth, which creates the illusion that the viewer is seeing objects in a virtual space [11]. This offers a number of immediate advantages to the researcher: greater control over stimulus presentation, variety in response options, and potentially increased ecological validity [12]. This has led to increased use of VR as a research tool across many psychological domains such as psychotherapy [13, 14], sports psychology [15], and social interaction [16].

The most apparent advantage of VR is the ability to present stimuli in three dimensions. This offers specific benefits depending on the research domain. For example, when discussing the potential application of VR to neuropsychological research, Rizzo et al. [17] describe virtual environments as “the ultimate Skinner box,” able to present a range of complex stimulus conditions that would not be easily controllable in the real world and enabling the examination of both cognitive processes (e.g., attention) and functional behaviours (e.g., planning and initiating a series of required actions). In clinical research VR is used to create complex scenarios, such as simulating exposure to a phobic stimulus, where the form and frequency of the exposure can be manipulated with absolute precision [4]. These examples highlight the difference between VR stimulus presentation and traditional experimental procedures: in VR the participant responds to pertinent stimuli while immersed in a larger virtual environment which can itself be controlled. This differs from traditional experimental contexts where the pertinent stimuli may be controlled but the surrounding environment often cannot be.

Of course, if VR were only a visual medium, then it could be argued that its only advantage over traditional experimental protocols is the ability to present visual stimuli along a third dimensional plane. However, as VR technology has advanced, many VR research studies now include varying levels and combinations of multimodal sensory input, allowing audio, haptic, olfactory, and motion to be experienced simultaneously to the graphically rendered environment or objects [18–20]. This greatly increases the user's sense of immersion in the virtual environment and allows the experimenter to create protocols that would not otherwise be possible. For example, exposure therapy is a common method employed in the treatment of anxiety disorders which, in the case of PTSD, may be difficult to implement for logistical or safety reasons. To overcome these issues, multimodal VR has been employed to create a virtual replica of a warzone, complete with audio and haptic feedback, to treat PTSD in war veterans [21, 22]. Where phenomena are known to occur due to a confluence of sensory data (e.g., audio and visual), multimodal VR enables the researcher to manipulate each input separately to gain a more accurate understanding of the relative contribution of each. For example, a recent study by Keshavarz et al. [23] employed this technique to assess the effects of auditory and visual cues on the perception of vection and resultant motion sickness in participants. Finally, multimodal environments are associated with faster mental processing times of discrete stimulus events, potentially because they provide the user with more complete information about the environment [24].

In addition to the presentation of experimental stimuli, VR enabled researchers to develop new protocols to measure participant responding. Many researchers have no doubt lamented the situation where studies that aim to assess a complex psychological construct (e.g., attention) have, out of necessity, been reduced to a mere “point and click” exercise for the participant. Most experiments strike a difficult balance between control and ecological validity, and very few replicate the multifaceted nature of real-life human responding [25]. It has been suggested that VR environments might help bridge this gap by allowing participants to respond in a manner that is more natural [26]. This can be seen across a range of psychological topics. For example, studies on altruism or prosocial behaviour are often carried out using hypothetical scenarios and self-report responses [27]. Kozlov and Johansen [2] on the other hand, employed a novel approach to examining this topic using VR. As participants attempted to navigate out of a virtual maze, under time pressure, virtual avatars approached the participant for help in a variety of situations. This enabled the experimenters to measure actual helping behaviours, as opposed to participants reporting what they would hypothetically do in such a situation. The researchers argue that even sophisticated high-level behaviours can be successfully examined using VR and suggest wider adoption. VR environments have also been used recently to examine the avoidance behaviour, a central component of fear that contributes to the maintenance of anxiety disorders. While many studies have examined the physiological and self-report aspects of fear, few have been able to examine the associated avoidance of, for example, the context or environment that elicits the fear response [28]. Glotzbach et al. [29] were able to directly examine avoidance behaviour by conditioning participants to be afraid of particular virtual environments and recording the extent to which they avoided returning to those environments later in the study. Finally, VR could be useful to measure responses in circumstances where it might be impractical or ethically questionable to do so in real life. For example, Renaud et al. [30] used a virtual environment and avatars to examine sexual affordances of convicted child molesters. The VR setup allowed the researchers to identify specific patterns of gaze behaviour exhibited by the experimental and not the control group of participants. They discuss a number of theoretical discussions that emerged from the study by virtue of the “first-person stance” enabled by using VR.

## 2. Questions about the Use of VR in Psychology Research

Since many of the advantages of VR as an experimental tool are derived from the ability to place the participant inside the scene, it is not surprising that a lot of research has been conducted into the concept of presence—the extent to which the user feels as though they are “really there” [31, 32]. Presence is viewed as crucial to having participants respond the same way in VR as they would in reality but remains a difficult concept to measure objectively [32–34]. Many studies have recorded user's subjective experience of presence and the perceived effect it has on engagement with tasks in a virtual environment [35–38]. Kober and Neuper [39] attempted to measure presence objectively and posit that it is characterised by increased attention toward stimuli in the virtual environment and correspondingly lower attention to VR irrelevant stimuli. They were able to identify distinct ERP patterns associated with increased presence. Furthermore, [32] found differences in the levels of presence elicited by a desktop VR system and a more immersive single-wall VR system, which was characterised by stronger activation of frontal and parietal brain regions, measured using EEG.

One of the determinants of presence is the level of immersion, described as the level of sensory fidelity offered by the VR system [40]. It has many contributing components such as field of view, field of regard, display size, and stereoscopy (not exhaustive) and although many use the terms presence and immersion interchangeably (e.g., [41]), they are very different concepts [31]. Immersion is an objective description of the technical capabilities of the VR system that describes the level of detail with which a virtual environment can be rendered, while presence describes the user's psychological response to said environment. Different users can experience different levels of presence in environment with the same level of immersion, depending on a range of factors such as state of mind. Still, it seems intuitive that a researcher would want higher levels of immersion wherever possible, as a higher-fidelity virtual world would elicit more generalizable responses. Indeed, immersive environments seem to be better remembered by participants [37], elicit more intense emotional responses [42], increase collaboration [43], and more successfully replicate the anxiety associated with real-life stressful situations [44]. At the same time, creating an environment elicits a sense of presence that is not entirely dependent on immersion. Factors such as personality and emotional state also influence presence [45–47]. In a research context, realism might not be determined by visual fidelity but by psychological fidelity: the extent to which stimulus presentation evokes the type of physiological or emotional response one would experience in real life. While immersion might help with this goal, it is not the only determining factor [3].

Indeed, it is not universally accepted that higher immersion is always better with some researchers reporting physical and psychological side effects from exposure to VR. These are collectively referred to as virtual reality-induced side effects (VRISE [48]) and often focus on a general feeling of malaise or perhaps motion sickness experienced by users [49]. The effect was initially believed to be caused by limitations in early VR technologies where there was often a lag between participant movements and the display being updated resulting in a disconnection between the perceptual and motor systems of the user [50]. However, while technological advances have overcome this early limitation, VRISE remain a problem [23, 51, 52]. Although common in most VR users, these side effects vary from person to person and, as such, it is difficult to pin down what aspects of immersion are responsible. While some studies suggest that more immersive HMDs are linked to higher levels of sickness in participants [53], others suggest that there is little difference between the side effects of using standard desktop computer display and a head-mounted VR display [54]. Regardless, it seems that these symptoms are generally mild and quick to subside and there is some evidence that users can adapt with repeated exposure [1, 55–57]. While not all that common in the literature, researchers should also consider potential psychological side effects of VR use, depending on the topic being examined. For example, Aardema et al. [58] found that users who had been exposed to an immersive virtual environment demonstrated increase in dissociative experience including a lessened sense of presence in objective reality as the result of exposure to VR, while Aimé et al. [59] found that VR immersion led to body dissatisfaction amongst users. As VR environments become more realistic and scenarios potentially more complex, another potential confound may arise from what Yee and Bailenson [60] term the Proteus effect, where users in a VR environment change their behaviour depending on how they are represented in the virtual world, though currently this effect seems limited to studies that use third-person view and avatars, as opposed to first person perspective [61].

## 3. The Use of VR in Visual Perception Research

In many domains, the benefits of VR stem from the ability to create recognisable, three-dimensional facsimiles of real objects in space. As a simplified example, let us imagine a study that asks participants to attend to the environment and respond every time they see a person with a happy face. Here the researcher needs only the object (face) to be presented, to be recognised by the participant, and to measure some level of reaction on the part of the participant. In such a context, the main technical focus in relation to VR is likely to be the visual fidelity of the stimuli—the extent to which the faces can be detailed enough for participants to distinguish their expressions. In the experimental study of visual perception, however, the researcher is concerned with how the stimuli are perceived. Here, VR offers both advantages and drawbacks when compared with real life and traditional experimental apparatus. In the following section, we focus on interesting aspects of immersive VR environments that impact how we examine perception: (1) space and movement and (2) tighter control over the visual scene.

### 3.1. The Effect of Space and Movement in VR on Perception

One area where complications arise is in the perception of space [62]. Many studies have observed a disparity between judgements of distance and perceptual actions such as reaching [63, 64]. In addition, it has been found that in VR, users consistently underestimate the size of the environment and distance to objects [65]. Although not always replicated (e.g., [66]), this effect has been found to be consistent with binocular and monocular vision [67], with varying field of view [68] and even when providing motion parallax and stereoscopic depth cues to the observer [69]. Bingham et al. [62] provide a useful explanation: what we see in VR as an object is actually a series of images mediated by a display. While the user's vision is focused on the series of images that make up the virtual object, the object itself appears in a different location. As a result, when the user is viewing the object, there is disconnect between accommodation (the fixed viewing distance between the user and the display) and convergence (the user's eyes converging on the virtual object), two processes that are inextricably linked in viewing objects in actual reality. Some studies have suggested that this effect is an issue of perception-action recalibration, while others suggest that walking through the virtual environment with continuous visual feedback is necessary to cause rescaling of the perceived space [70].

On the other hand, there are instances where the disconnection between virtual and actual reality provides opportunities for the examination of perception, which would otherwise not be possible. Mast and Oman [71] used a virtual environment to examine visual reorientation illusions, a phenomenon reported by astronauts where the perceived identity of a surface is changed due to rotation of the entire visual field. This phenomenon is difficult to replicate in real life, as we are surrounded by visual cues in our environment that help us to orient ourselves (e.g., trees grow upwards), as well as the orienting force of gravity, which provides a consistent cue for “down.” The authors created an immersive environment (i.e., a room containing various objects) with intentionally ambiguous visual cues so that, due to the placement of objects in the room, it could appear correctly oriented even if the room were rotated by 90°. The researchers were then able to rotate the entire visual scene and examine the effects on perception—something that would be almost impossible to replicate in the physical environment.

In addition to creating new illusions, an immersive environment offers the possibility to examine commonly employed visual illusions in new contexts. Traditionally, illusions to examine perception are designed and employed assuming a stationary point of view and have not been studied thoroughly for a moving observer. By employing an immersive environment it is possible to investigate whether these illusions persist when the observer moves. This would be difficult to carry out using a two-dimensional computer screen setup, due to the fact that the stimuli, and hence the illusion, require the observer to view the screen head on. Bruder et al. [72] introduced the use of VR to investigate how visual motion illusions are perceived for a moving observer. The authors manipulated the optical flow—the change of the light pattern on the observer's eyes when moving in the environment—and found that optic flow manipulation can significantly affect users' self-motion judgments.

Movement can also add ecological validity to the examination of everyday perceptual phenomena. Change blindness, a phenomenon in which changes occurring in a visual scene are not noticed by the observer, occurs in a variety of contexts and its impact is studied in range of applied settings from courtroom eye-witness testimony to driving behaviour [73, 74]. Experimental examinations of the effect are usually done on a computer screen where two similar images are presented one after the other with a short blanking between the two, and observers have to indicate whether the second image is the same as the first one or if a change has occurred [75]. Using VR to create a more ecologically valid examination of the phenomenon, Suma et al. [76] had the observer walk through an immersive virtual environment and found that even large changes in the surrounding environment were unlikely to be noticed.

### 3.2. Control over the Visual Scene

Virtual reality technology overcomes a number of the limitations of traditional experimental methods by enabling precise control of the spatial distribution of the light in the visual scene as well as distance and position of stimuli. In a real room, it is not possible to manipulate these elements completely independently. However, with virtual reality it is possible to manipulate the distances between the surfaces whilst at the same time maintaining the same photometric relationships (i.e., the amount of light reaching the observers' eyes remained constant). Furthermore, by manipulating objects in three-dimensional space, it is possible to examine the effects of positive and negative parallax which would not be possible using a two-dimensional screen. Moreover, the VR technology allows full control of the amount of light reaching the observers' eyes and of the spatial arrangement of the surfaces in the visual scene.

This level of control is particularly useful when we examine colour perception and particular visual phenomena such as colour contrast phenomenon [77]. The colour contrast phenomenon refers to the condition whereby two surfaces with the same spectral composition are perceived to have a different colour when they are placed against different chromatic backgrounds. It has been shown that this phenomenon depends on perceptual belongingness, the grouping of a set of apparent elements into a perceived whole [78, 79]. As Gilchrist et al. [80] explained, “When the [contrast] display is presented in a textbook, it is perceived to belong to the page of the book and to the table on which the book is lying. Thus, […] the illusion should be quite weak” (p. 814). Adopting a VR technology prevents surfaces from outside of the experimental display from affecting the experimental examination of the colour contrast phenomenon. Although the contrast phenomenon has been a focus of centuries of debate that has interested scientists and philosophers since Aristotle's time [81], there is still no shared consensus of why it actually happens as some authors attribute its occurrence to high-level factors of the visual process whilst others claim that the phenomenon is due to low-level factors. In an attempt to disentangle these viewpoints, Soranzo et al. [82] studied this phenomenon in VR and provided evidence that the colour contrast phenomenon may be attributed to the summative effect of factors occurring to both high- and low-level factors of the visual process.

## 4. Conclusions

The proliferation of available virtual reality (VR) tools has seen increased use in experimental psychology settings over the last twenty years. In this review, we outlined the advantages and disadvantages of this technology in psychological research, compared to more traditional apparatus. The advantages of VR are that it allows greater control over stimulus presentation; variety in response options; presentation of stimuli in three dimensions; the creation of complex scenarios; the generation of varying levels and combinations of multimodal sensory input potentially allowing audio, haptic, olfactory, and motion to be experienced simultaneously to the graphically rendered environment or objects; the possibility for participants to respond in a more ecologically valid manner; the precise and independent manipulation of the geometric and photometric relationships between objects; the possibility of examining sophisticated complex participants behaviours, such as avoidance; and the study of situations which can be impractical, dangerous, or ethically questionable to be created in real life.

Additionally, we suggest that although this technology has enormous potential to facilitate new discoveries in psychology, there are certain variables that need to be taken into account by the researcher including the concept of presence—immersion alone is not necessarily sufficient to make the participant feel as if the virtual objects are “really there” and respond accordingly; physical and psychological side effects from exposure to VR (virtual reality-induced side effects). In addition, we considered issues that emerge from use of VR in the examination of visual perception and how comparative differences in the perception of colour, contrast, space, and movement, when compared to real life, can be a concern if the goal is exact replication of perception in the physical world or an advantage when trying to create “impossible scenarios.”

Finally, it is worth noting that there are large variations in the size and cost of the various apparatus and in some cases they can be impractical for some settings due to their technological complexities. Until quite recently, the price of immersive HMDs with a good tracker system could be prohibitive. However, HMDs are now becoming cheaper and easier to obtain [83, 84], while virtual reality caves, for example, are still comparatively more expensive and require a large amount of space to install [9, 85]. Nevertheless, VR offers exciting opportunities and we hope to see future work that more thoroughly examines the psychometric properties of this useful research tool.
